# Supplementary material for: Record high room temperature resistance switching in ferroelectric-gated Mott transistors unlocked by interfacial charge engineering
Source: Nat Commun. 2023 Dec 12;14:8247. doi: 10.1038/s41467-023-44036-x (PMC10716183; doi:10.1038/s41467-023-44036-x)
Supplement: Supplementary file 1 — Supplementary Information [file 41467_2023_44036_MOESM1_ESM.pdf]

Record High Room Temperature Resistance Switching in Ferroelectric-Gated Mott Transistors Unlocked by Interfacial Charge Engineering (Supplementary Information)

Yifei Hao[1,7], Xuegang Chen[1,7], Le Zhang[1], Myung-Geun Han[2], Wei Wang[2], Yue-Wen Fang[3,4], Hanghui Chen[5,6], Yimei Zhu[2], and Xia Hong[1*]

[1] Department of Physics and Astronomy & Nebraska Center for Materials and Nanoscience, University of Nebraska-Lincoln, Lincoln, Nebraska, 68588-0299, USA

[2] Condensed Matter Physics and Materials Science, Brookhaven National Laboratory, Upton, New York 11973-5000, USA

[3] Fisika Aplikatua Saila, Gipuzkoako Ingeniaritza Eskola, University of the Basque Country (UPV/EHU), Europa Plaza 1, 20018 Donostia/San Sebastián, Spain

[4] Centro de Física de Materiales (CSIC-UPV/EHU), Manuel de Lardizabal pasealekua 5, 20018 Donostia/San San Sebastián, Spain

[5] NYU-ECNU Institute of Physics, NYU Shanghai, Shanghai 200062, China

[6] Department of Physics, New York University, New York 10002, USA

[7] These authors contributed equally: Yifei Hao, Xuegang Chen

[*] Email: X.H. (xia.hong@unl.edu)

Supplementary Note 1: Structural Characterization

For bulk samples, $LaNiO_3$ has the rhombohedral structure ($R\overline{3}c$), while $NdNiO_3$ and $SmNiO_3$ are orthorhombic in the metallic phase ($pbnm$)[1]. Supplementary Fig. 1 shows the x-ray diffraction (XRD) θ-2θ spectra taken on correlated oxide and ferroelectric thin films and heterostructures, which reveal single crystalline growth with no impurity phases. The c-axis lattice constants of single-layer $LaNiO_3$ (LNO) and $NdNiO_3$ (NNO) films on (001) $LaAlO_3$ (LAO) substrates are 3.88 Å and 3.81 Å, respectively. The c-axis lattice constants of single-layer $La_{0.67}Sr_{0.33}MnO_3$ (LSMO) and $PbZr_{0.2}Ti_{0.8}O_3$ (PZT) films on (001) $SrTiO_3$ (STO) substrates are 3.85 Å and 4.19 Å, respectively. Supplementary Fig. 1c shows the XRD data taken on a PZT/$NdNiO_3$ heterostructure deposited on (001) LAO substrate. Supplementary Fig. 1f shows the XRD data taken on a PZT/$LaNiO_3$/LSMO heterostructure deposited on (001) $SrTiO_3$. For the heterostructures, the signals for the ultrathin-correlated oxide layers are too small to be detected. High-resolution scanning transmission electron microscopy (HRSTEM) measurements confirm the high crystallinity and atomically sharp interfaces of the samples (Supplementary Fig. 2). The growth

rates of films are calibrated using x-ray reflectivity measurements, which is consistent with the HRSTEM result.

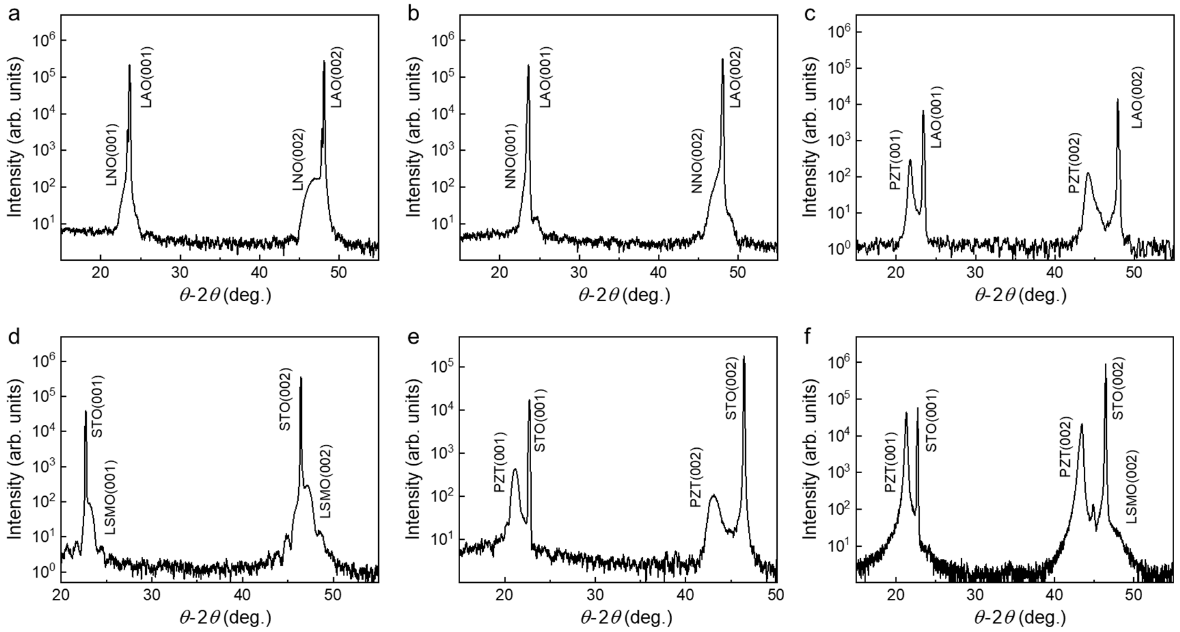

Supplementary Fig. 1 | Structural characterization. XRD θ-2θ scans taken on **a** 5 nm LNO on LAO, **b** 6 nm NNO on LAO, **c** 150 nm PZT/6.5 unit cell (uc) NNO heterostructure on LAO, **d** 10 nm LSMO on STO, **e** 16 nm PZT on STO, and **f** 100 nm PZT/3 uc LNO [LNO(3)]/LSMO(2) heterostructure on STO.

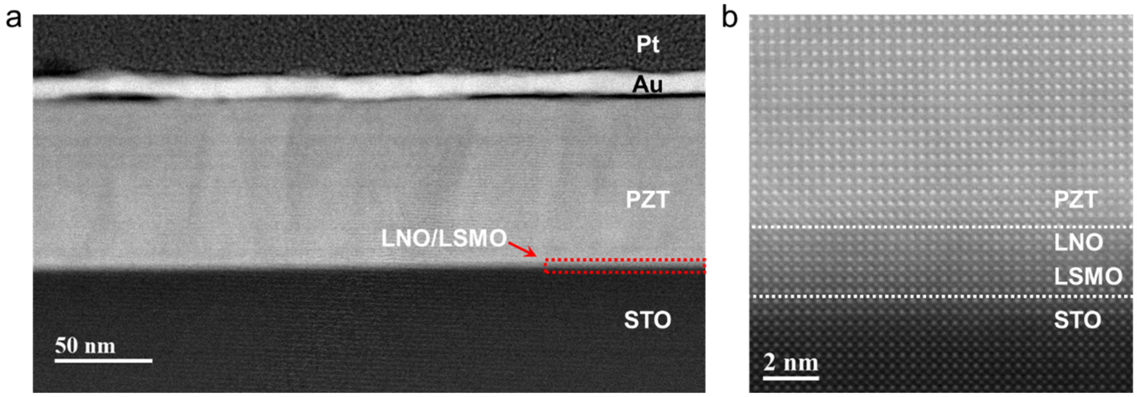

Supplementary Fig. 2 | TEM characterization. Cross-sectional HRSTEM image of the PZT/LNO(3)/LSMO(3) sample shown in Fig. 1c in the main text at different magnifications. The ultrathin LNO/LSMO layers are relatively invisible in **(a)** with a shadowed grey color, as highlighted in the red box.

We have also analyzed the Mn valence state via electron energy loss spectroscopy (EELS). Supplementary Fig. 3 shows the EELS data of Mn *L* edge obtained on the PZT/LNO(3)/LSMO(3) sample shown in Fig. 1c-d in the main text. The Mn *L* spectra are observed within 2 uc above and below the interfaces of LSMO (Supplementary Fig. 3b,c). The broadening of the EELS signal can be caused by the intermixing across the interface (Mn diffusion) and atomic scale interface terraces, as well as core-loss signal delocalization, electron probe dechanneling due to the atomic distortion near/at the interface, and multiple scattering[2]. We extract the position and intensity of Mn $L_{2,3}$ peaks via Lorentzian fitting (Supplementary Fig. 3d), from which we analyze the interfacial charge transfer effect. The energy shift of the Mn L_3 edge and the L_3/L_2 intensity ratio are shown in Fig. 4f in the main text. Note that the L_3/L_2 intensity ratio obtained from the Lorentzian fitting can be slightly underestimated compared to analysis based on a double-arctangent background model.

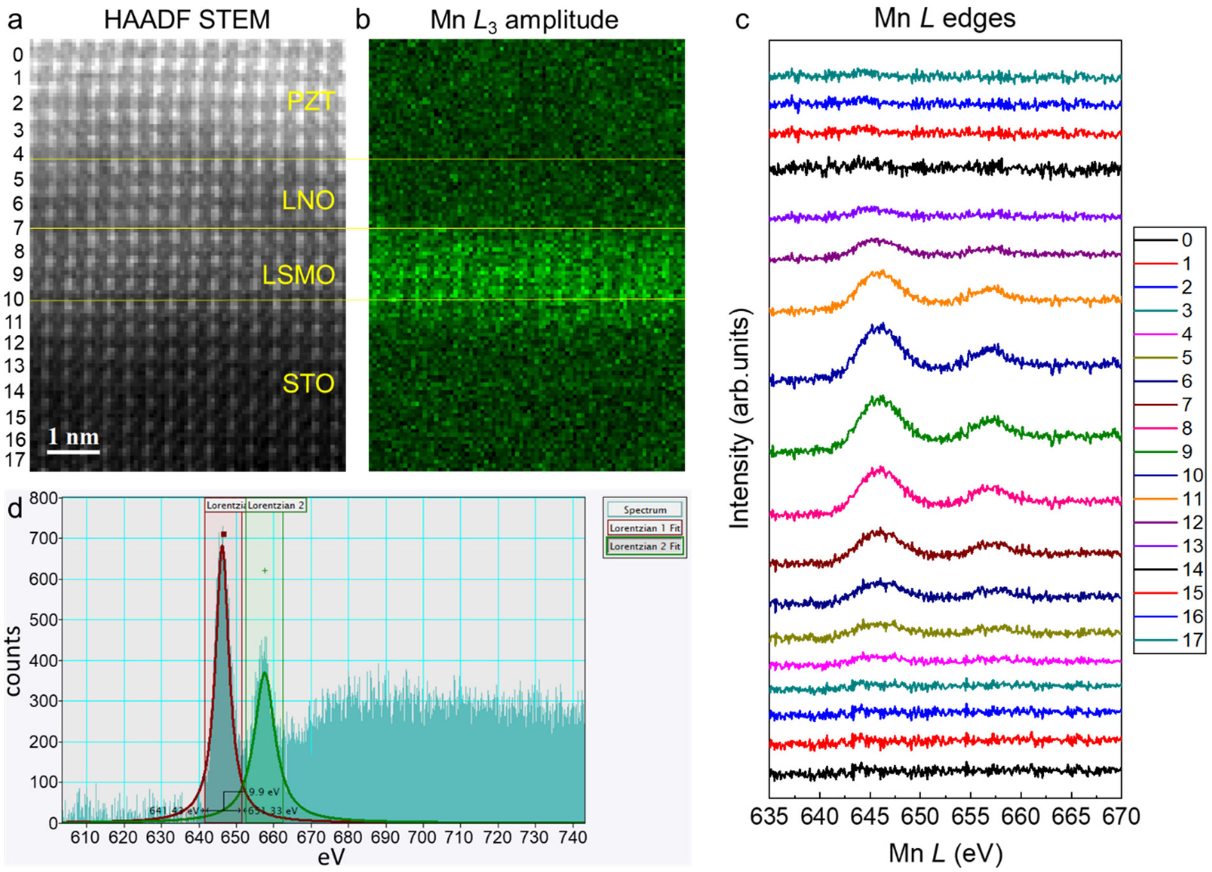

Supplementary Fig. 3 | EELS Mn spectra. a-c Cross-sectional HRSTEM image **(a)**, EELS mapping of Mn element **(b)**, and Mn *L* edge spectra **(c)** taken on the PZT/LNO(3)/LSMO(3) heterostructure. **d** One example of Lorentzian fitting to Mn $L_{2,3}$ peaks of unit cell #9.

Supplementary Note 2: Device Fabrication and Characterization

Supplementary Fig. 4 shows the *P-E* loop on an Au/PZT/10 nm LNO capacitor device. We apply a triangular wave of bias voltage to the capacitor and measure the switching current as a function of time. The polarization is calculated by the time integral of the switching current. The detailed method is discussed in Ref. [3]. From the hysteresis, we obtain a remanent polarization of 77 μC cm^{-2} for PZT.

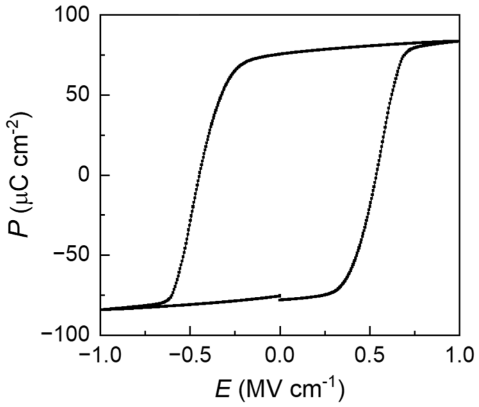

Supplementary Fig. 4 | Polarization vs. electric field hysteresis of PZT. *P*(*E*) loop taken on an Au/100 nm PZT/10 nm LNO capacitor.

Supplementary Fig. 5 shows the process flow for the fabrication of the field effect transistor (FET) devices. We first define Hall bar devices with different channel dimensions using photolithography (Supplementary Fig. 5a) and deposit about 15-20 nm Ti on top of the entire substrate (Supplementary Fig. 5b). The sample is then sonicated in acetone for a couple of seconds followed by IPA rinsing to remove the photoresist. The Ti layer deposited on the photoresist is removed during this lift-off process. The patterned substrate is annealed at 400 °C in a muffle oven for 4 hours, with the Ti layer oxidized into amorphous TiO_x and becoming transparent (Supplementary Fig. 5c). Epitaxial oxide thin films can only form in the Hall bar area, as the rest area of the substrate is covered by amorphous TiO_x. After *in situ* deposition of ultrathin correlated oxide layers and thick PZT films on the patterned substrate (Supplementary Fig. 5d), we perform the second photolithography to define electrodes for the Hall bar device and top gate (Supplementary Fig. 5e). Scratches are made in the Hall bar electrode area to ensure connection of the Au contact to the correlated channel. After Au deposition, we perform a second lift-off to remove the photoresist and the Au layer on top (Supplementary Fig. 5f).

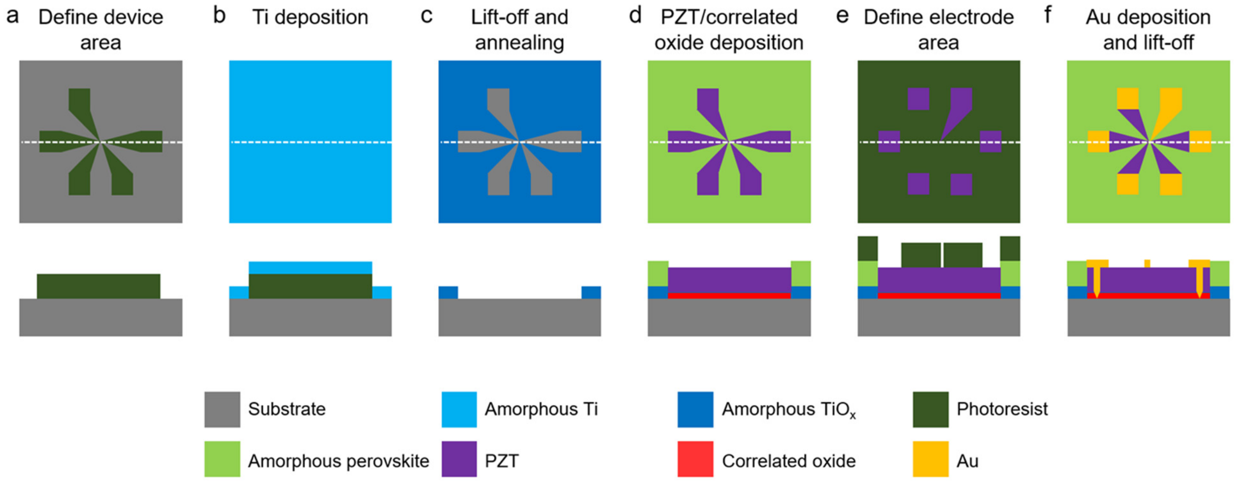

Supplementary Fig. 5 | Schematic device fabrication flow. a First photolithography to define device area. **b** Ti deposition. **c** Lift-off and annealing. **d** Thin film deposition. **e** Second photolithography to define electrode area. **f** Au deposition and lift-off.

Supplementary Fig. 6 shows the Hall resistivity ρ_{xy} vs. magnetic field taken on a 4 uc $LaNiO_3$ thin film sample (Supplementary Fig. 6a), and the on (Supplementary Fig. 6b) and off (Supplementary Fig. 6c) states of a PZT/LNO(4) sample. We deduce the Hall coefficient $R_H = 1/ne$ from the slope of $\rho_{xy}(B)$ and calculate the 3D carrier density n to be 5.40×10^{22} cm^{-3} for the 4 uc film, consistent with the theoretically predicted value[4]. For the PZT/LNO(4) sample, n is 5.36×10^{22} cm^{-3} for the on state and 4.73×10^{22} cm^{-3} for the off state, yielding a polarization field of about 76 μC cm^{-2} for PZT, which is consistent with the P-E loop data (Supplementary Fig. 4).

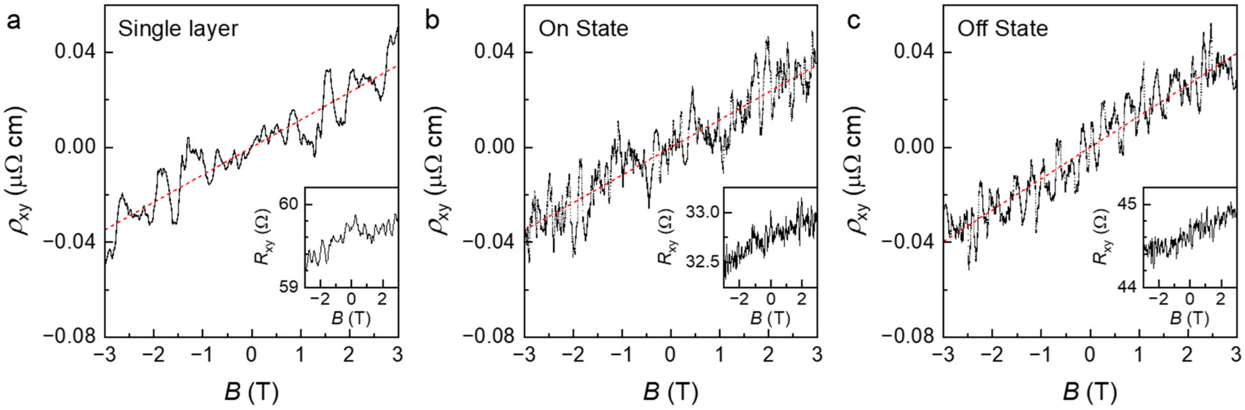

Supplementary Fig. 6 | Hall effect measurement taken on 4 uc $LaNiO_3$ and PZT/LNO(4) samples. Asymmetrized ρ_{xy} vs. B taken on a 4 uc $LaNiO_3$ film **(a)**, and the on **(b)** and off **(c)** states of a PZT/LNO(4) device with linear fits (dashed lines). The insets show the $R_{xy}(B)$ data.

Supplementary Note 3: Ferroelectric Field Effect Switching in Single-Layer $LaNiO_3$ Channels

We observe robust resistance switching hysteresis in PZT-gated single-layer $LaNiO_3$ channels. Supplementary Fig. 7 shows the room temperature resistance switching data taken on 2-10 uc $LaNiO_3$ single-layer channels. The resistance switching ratio for the LNO(2) device is $\Delta R/R_{on}$ = 1,619%, which is the highest value for Mott FeFETs with single-layer correlated oxide channels reported to date.

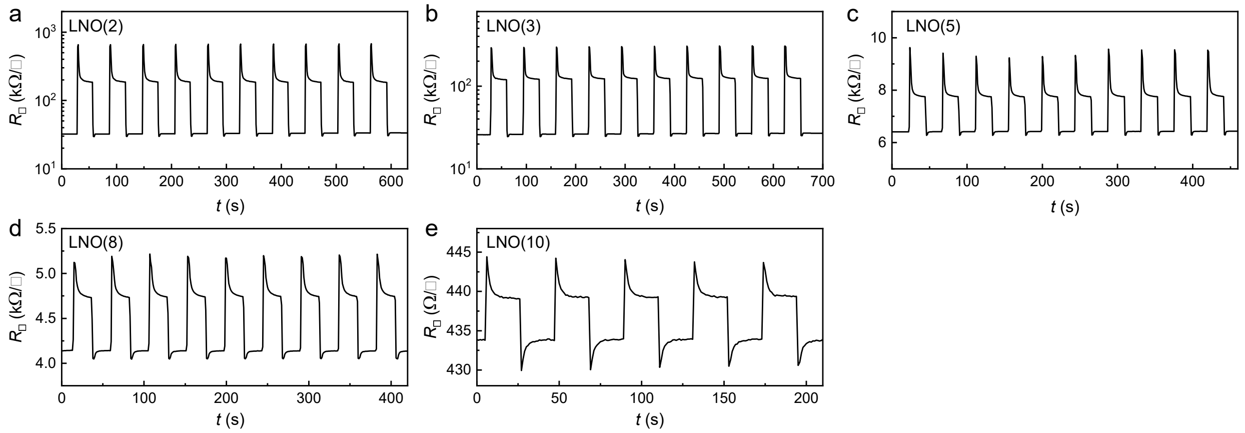

Supplementary Fig. 7 | Ferroelectric field effect at 300 K in single-layer LNO channels. Resistance switching upon applied voltage pulses with opposite polarities taken on single-layer channels: **a** LNO(2), **b** LNO(3), **c** LNO(4), **d** LNO(8), and **e** LNO(10).

Supplementary Note 4: Ferroelectric Field Effect Switching in Single-Layer $NdNiO_3$ Channels

Supplementary Fig. 8 shows the room temperature resistance switching data taken on 2.5-9 uc $NdNiO_3$ single-layer channels. The highest $\Delta R/R_{on}$ is 121% observed in the 2.5 uc $NdNiO_3$ device. Rapid decay in the off-state resistance has been observed in the 2.5 uc and 3 uc samples, which can be attributed to the uncompensated depolarization field as the $NdNiO_3$ layer thickness is below the electric dead layer thickness.

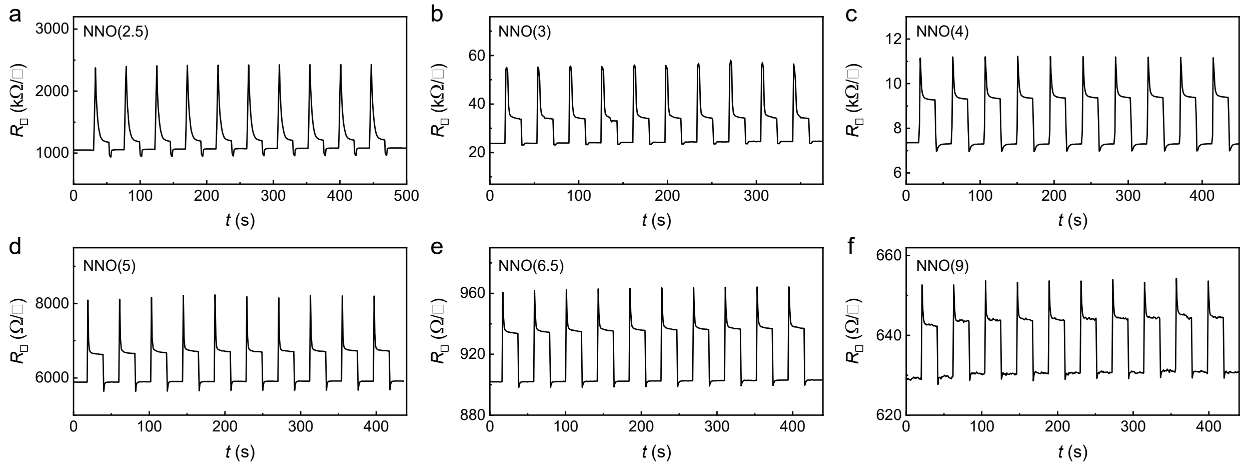

Supplementary Fig. 8 | Ferroelectric field effect at 300 K in single-layer NNO channels. a-f, Resistance switching upon applied voltage pulses with opposite polarities taken on single-layer channels: **a** NNO(2.5), **b** NNO(3), **c** NNO(4), **d** NNO(5), **e** NNO(6.5), and **f** NNO(9).

Supplementary Note 5: Ferroelectric Field Effect Switching in Single-Layer $SmNiO_3$ Channel

The ferroelectric field effect in the $SmNiO_3$ channel is weak due to the lack of itinerant charge carriers. Supplementary Fig. 9 shows the resistance switching data taken on the 8 uc $SmNiO_3$ single-layer channel, which yields $\Delta R/R_{on}$ of 1.1%.

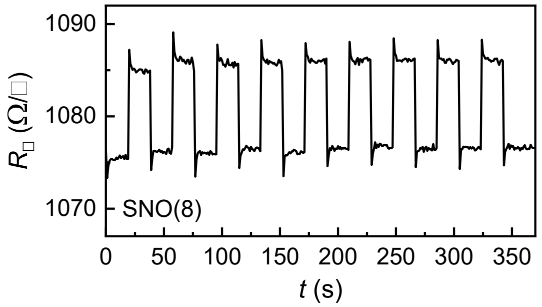

Supplementary Fig. 9 | Ferroelectric field effect at 300 K in single-layer SNO channel. Resistance switching upon applied voltage pulses with opposite polarities taken on the single-layer SNO(8) channel.

Supplementary Note 6: Ferroelectric Field Effect Switching in $LaNiO_3/La_{0.67}Sr_{0.33}MnO_3$ Bilayer Channels

We observe robust resistance switching hysteresis in PZT-gated $LaNiO_3/La_{0.67}Sr_{0.33}MnO_3$ bilayer channels (Supplementary Fig. 10).

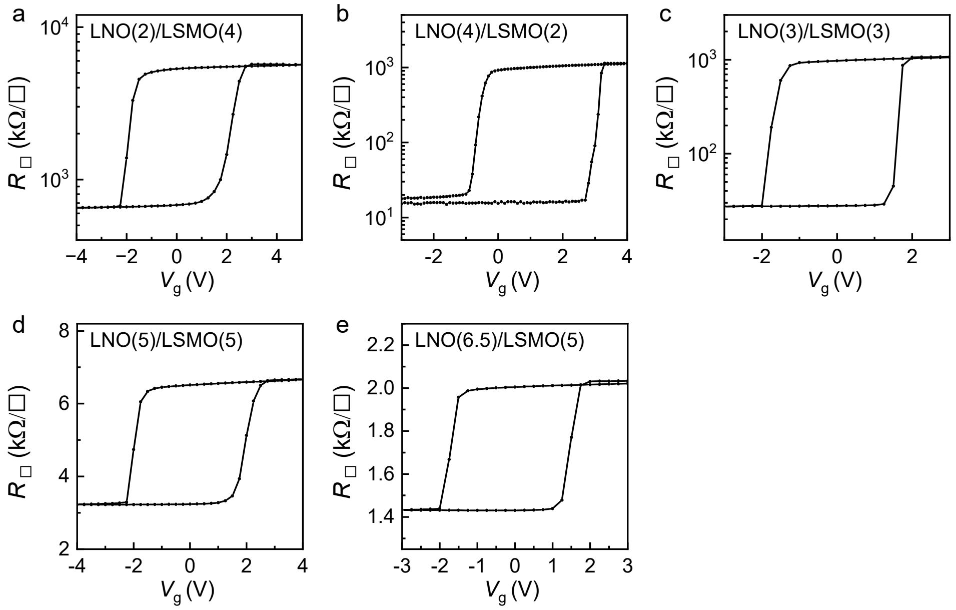

Supplementary Fig. 10 | Resistance switching hysteresis at 300 K in bilayer LNO/LSMO channels. $R_{\square}$ vs. V_g switching hysteresis taken on bilayer channels: **a** LNO(2)/LSMO(4), **b** LNO(4)/LSMO(2), **c** LNO(3)/LSMO(3), **d** LNO(5)/LSMO(5), and **e** LNO(6.5)/LSMO(5).

Supplementary Fig. 11 shows the resistance switching taken on a series of $LaNiO_3/La_{0.67}Sr_{0.33}MnO_3$ bilayer channels with different layer thickness combinations.

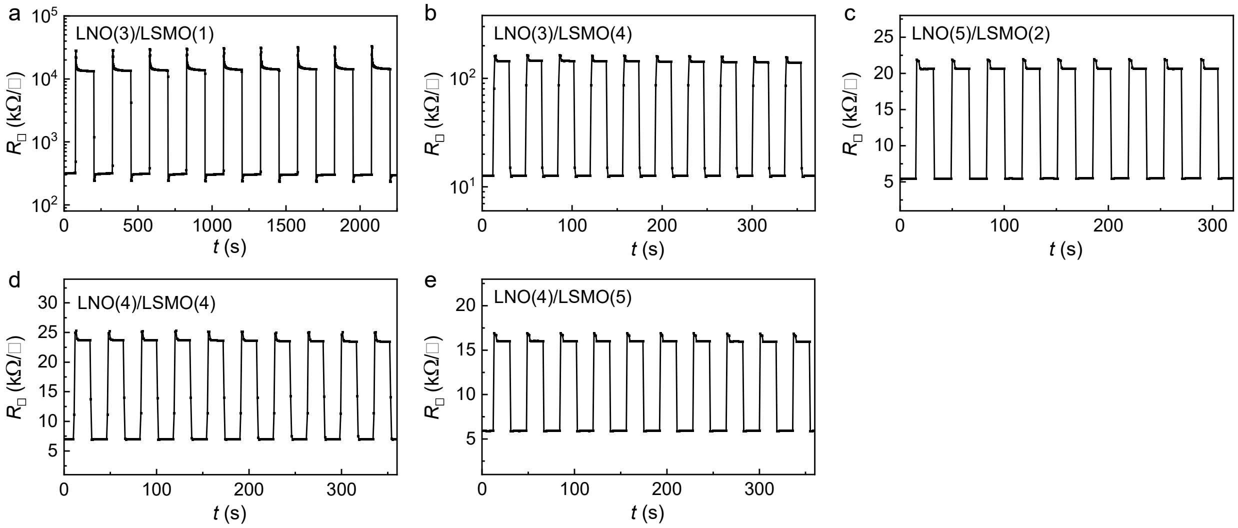

Supplementary Fig. 11 | Ferroelectric field effect at 300 K in bilayer LNO/LSMO channels. a-e, Resistance switching upon applied voltage pulses with opposite polarities taken on bilayer channels: **a** LNO(3)/LSMO(1), **b** LNO(3)/LSMO(4), **c** LNO(5)/LSMO(2), **d** LNO(4)/LSMO(4), and **e** LNO(4)/LSMO(5) channels.

Supplementary Note 7: Ferroelectric Field Effect Switching in $NdNiO_3/La_{0.67}Sr_{0.33}MnO_3$ Bilayer Channels

We observe robust resistance switching hysteresis in PZT-gated $NdNiO_3/La_{0.67}Sr_{0.33}MnO_3$ bilayer channels (Supplementary Fig. 12). The resistance switching ratio for the $NdNiO_3(4)/La_{0.67}Sr_{0.33}MnO_3(4)$ bilayer channel is $\Delta R/R_{on}$ ~1,225%.

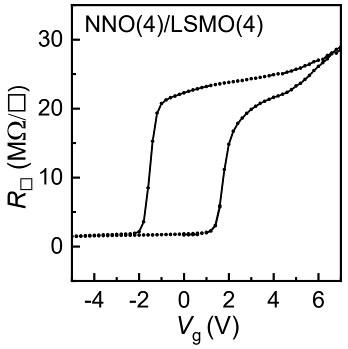

Supplementary Fig. 12 | Ferroelectric field effect at 300 K in bilayer NNO/LSMO channel. $R_\square$ vs. V_g switching hysteresis taken on NNO(4)/LSMO(4) bilayer channel.

Supplementary Fig. 13 shows the resistance switching data taken on a series of $NdNiO_3/La_{0.67}Sr_{0.33}MnO_3$ bilayer channels with different layer thickness combinations. The resistance switching ratio for the $NdNiO_3(4)/La_{0.67}Sr_{0.33}MnO_3(2)$ bilayer channel is $\Delta R/R_{on}$ ~5,319%.

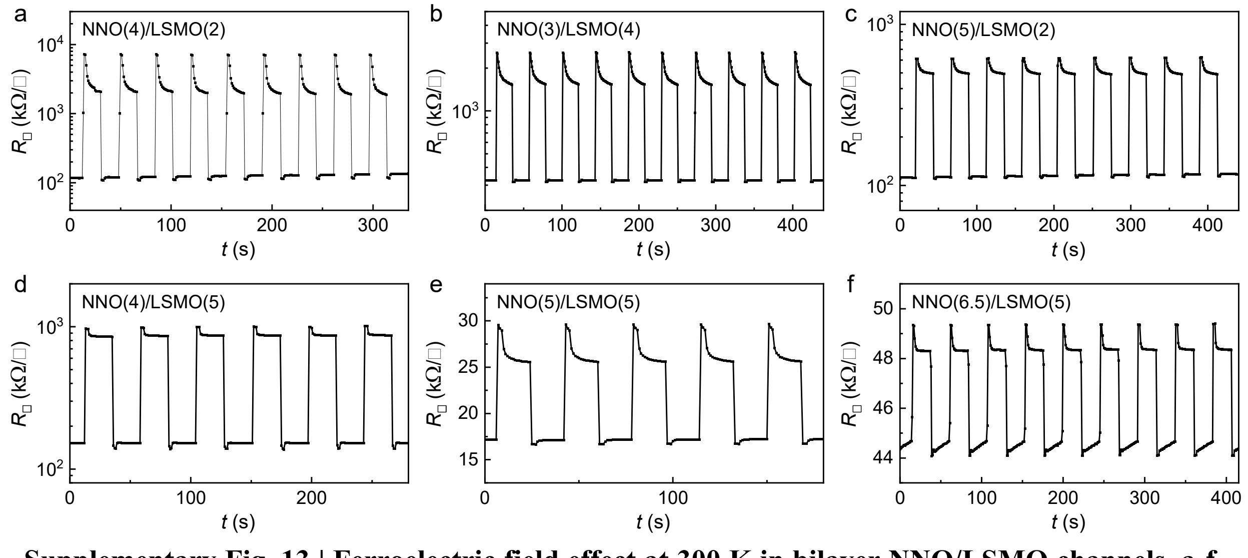

Supplementary Fig. 13 | Ferroelectric field effect at 300 K in bilayer NNO/LSMO channels. a-f, Resistance switching upon applied voltage pulses with opposite polarities taken on bilayer channels: **a** NNO(4)/LSMO(2), **b** NNO(3)/LSMO(4), **c** NNO(5)/LSMO(2), **d** NNO(4)/LSMO(5), **e** NNO(5)/LSMO(5), and **f** NNO(6.5)/LSMO(5).

Supplementary Note 8: DFT+*U* Calculation of $NdNiO_3/La_{0.75}Sr_{0.25}MnO_3(LaMnO_3)$ Superlattices

Supplementary Fig. 14 shows the DFT+*U* calculations of the optimized crystal structure of $NdNiO_3(4)/La_{0.75}Sr_{0.25}MnO_3(4)$ (Supplementary Fig. 14a) and $NdNiO_3(4)/LaMnO_3(4)$ (Supplementary Fig. 14b) superlattices, along with the site-resolved Mn or Ni 3*d* magnetic moments. The corresponding charge transfer is shown in Fig. 4e in the main text.

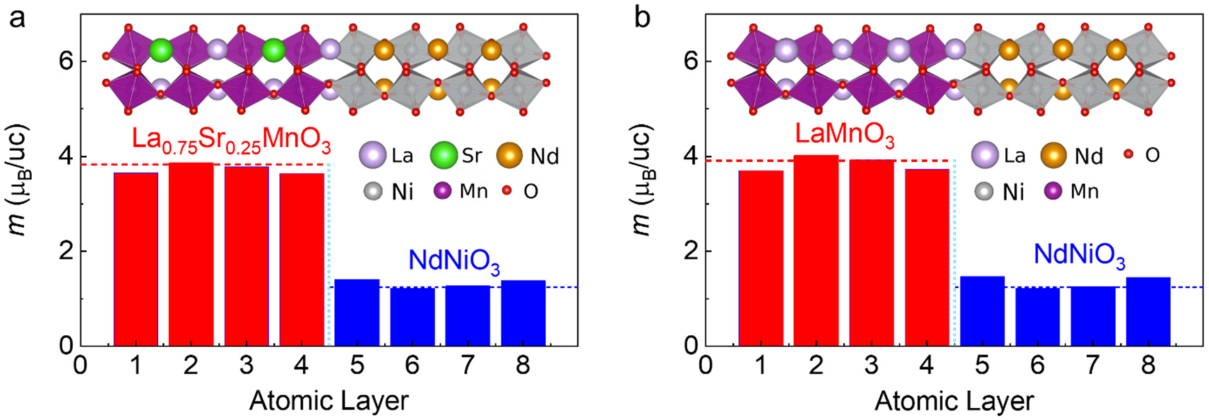

Supplementary Fig. 14 | DFT+*U* calculation of $NdNiO_3(4)/La_{0.75}Sr_{0.25}MnO_3(4)$ and $NdNiO_3(4)/LaMnO_3(4)$ superlattices. Atomic layer projected Mn/Ni magnetic moments of **a** $NdNiO_3(4)/La_{0.75}Sr_{0.25}MnO_3(4)$ and **b** $NdNiO_3(4)/$ $LaMnO_3(4)$ superlattices. The red and blue dashed lines represent the Mn/Ni magnetic moments of DFT-calculated bulk $La_{0.75}Sr_{0.25}MnO_3$ ($LaMnO_3$) and $NdNiO_3$, respectively. Insets: Schematics of the supercells.

References

1 S. Catalano, M. Gibert, J. Fowlie, J. Íñiguez, J. M. Triscone, and J. Kreisel, Rare-earth nickelates *R*NiO$_3$: thin films and heterostructures, Reports on Progress in Physics **81**, 046501 (2018).

2 K. Kimoto, T. Asaka, T. Nagai, M. Saito, Y. Matsui, and K. Ishizuka, Element-selective imaging of atomic columns in a crystal using STEM and EELS, Nature **450**, 702 (2007).

3 Y. Hao, T. Li, Y. Yun, X. Li, X. Chen, J. Song, Z. Ahmadi, J. E. Shield, X. Xu, and X. Hong, Tuning Negative Capacitance in $PbZr_{0.2}Ti_{0.8}O3/SrTiO_3$ Heterostructures via Layer Thickness Ratio, Physical Review Applied **16**, 034004 (2021).

4 J. Fowlie, M. Gibert, G. Tieri, A. Gloter, J. Íñiguez, A. Filippetti, S. Catalano, S. Gariglio, A. Schober, M. Guennou, et al., Conductivity and Local Structure of $LaNiO_3$ Thin Films, Advanced Materials **29**, 1605197 (2017).
